# Some professional experiences

**DOI:** 10.4103/0019-5545.43629

**Published:** 2008

**Authors:** C. Shamasundar

**Affiliations:** 250, 43^rd^ Cross, 9^th^ Main, 5^th^ Block, Jayanagar, Bangalore - 560 041, India

**Keywords:** Diagnosis, schools of psychotherapy, teaching-learning, methods and techniques, Indian mythology

## Abstract

This narrative of some of my professional experiences attempts to present circumstances in which I arrived at certain conclusions like: (a) Subjective personal experiences can be valid contributors to integration of a professional's world-view. (b) Concepts and constructs relating to diagnosis and other systems of understanding and practice are useful only as reference-systems. Rigidity in their use can be counter-productive. (c) Mental health profession is not always indispensable to patient's well being. Professional pride is an obstacle to learning. (d) Psychotherapy is not consciously done, but is a natural consequence of therapist's efforts to understand the patient and his/her predicament. Therapist's qualities play a predominant role. (e) Usefulness of therapeutic methods and techniques is dependent entirely on how the therapist uses them. (f) Absolute therapeutic effectiveness is a myth. (g) Indian mythology can offer a system of “Psycho-pathology,” a system of explanation and management, parallel to existing systems.

## INTRODUCTION

This article narrates a few of my experiences and their effects on my understanding, selected for their novelty. Some of NIMHANS alumni may remember having heard bits and pieces of these experiences. The experiences profoundly influenced my clinical conduct by: (i) affecting the manner of my ‘professional’ cognition, (ii) prompting me to develop few constructs or concepts for bringing about some semblance of holism to my understanding, (iii) contributing to gradual emergence of a few ideas as principles of therapy that I try to adapt in my therapeutic work. I suspect that most fellow professionals would have encountered similar experiences and arrived at similar or different conclusions. After all, variability is a characteristic of natural phenomena in every dimension of its manifestation. If we can pool such experiences, some principles may eventually emerge, common to most professionals. Consequently, I hope that the following narrative will prompt the reader to reflect on them. I expect that, in the long run, investigative-psychiatry will accord equal importance to subjective experiences and their possible validity, leading to enrichment of psychotherapeutic literature.

## ‘SCIENTIFIC PRECISION,’ OR PROBABILITIES AND ‘SPECTRUMS’?

I followed-up same patients for over 3½ years while working at Napsbury Hospital near St. Albans (UK) from 1971 to mid 1974. Many of them were readmitted twice or thrice. Nearly one third of them used to require review of diagnosis and its change. For example, a functioning obsessive-compulsive neurotic would become a dysfunctional paranoid schizophrenic a year later. Switchover from a neurotic to an affective disorder and vice-versa was most common. These changes occurred in spite of elaborate and repeated clinical assessments. Similarly, I found that the more and more I interacted with the patient, my understanding about that patient kept on evolving.

For me, this inconsistency of diagnosis was a rude shock. The effect was, ***I lost absolute faith in diagnosis.** I do not mean diagnosis is invalid or un-necessary. The profession cannot clinically and academically function without the instrument of diagnosis, which represents a set of commonly agreed upon concepts and constructs for professional convenience of understanding and communication*. Diagnosis represents a tentative assumption about the probable clinical state of a patient at a given point in time. Like the latitudes and longitudes, the underlying concepts and constructs serve as a reference-grid to infer a workable meaning from the clinical data. Thus, when two or more professionals with different ‘reference-grids’ were to read through recorded clinical histories, they are likely to deduce different diagnosis. But, we cannot expect the patient's psychopathology, un-manifest and manifest to be according to our scheme of things.

*Thus, our diagnostic constructs need not and do not always correspond to what is happening in the patient. As mentioned earlier, their best use is only as reference-points and conceptual templates for the convenience of understanding and communication*. Giving them any importance beyond these purposes will jeopardize the efficient management of patient, by distracting and diluting our conscious and un-conscious involvement with *real problems* that require attention or even masking the problems. About a decade later, the concept of ‘spectrum of disorders’ began to appear in professional literature.

Excessive importance to diagnosis is an obstacle in the field of psychotherapy.

## ‘SCHOOLS’ OF PSYCHOTHERAPY, OR JUST DIFFERENT PERSPECTIVES?

While at Napsbury, I simultaneously worked for three consultants, looking after some of their patients. One was a senior Jungian training analyst, another was a disciple of Maxwell Jones and an expert in therapeutic community, and the like. The other was an eclectic. We used to conduct therapeutic ward-group meetings twice a week in a ‘sub-acute’ ward, and once a week in an ‘acute ward.’ My psychotherapy supervisor was a Kleinian analyst. In that hospital, a clinical psychologist colleague of mine was undergoing training in Freudian analytical group psychotherapy. Upon my request, his supervisor permitted me to participate in the group-sessions as a silent participant. After over a year, this privilege was terminated, and I never came to know what complications, if any, in the group process or in the trainee-therapist led to this decision.

Possibly, as a consequence of this exposure to many ‘schools’ and modalities, ***I lost absolute faith in ‘schools of psychotherapy.’** The ‘schools of psychotherapy’ are not fiction. On the contrary, they are valid, as I have been able to observe most of the described concepts in my clinical work. All of them are equally valid from respective ‘reference points.’* For example, suppose that two psychiatrists believe in two different sets of concepts. If they happen to read through a detailed case history, each will ‘see’ evidences for one's concepts. Also, in my patients, I have been able to understand different parts of pathology by applying concepts from different schools. As mentioned above in respect of diagnosis, we cannot expect the patient and his/her family system to develop psychopathology according to our concepts and constructs. This is the reason for emergence of so many schools of psychotherapy to make some workable sense of the extreme variability in clinical manifestation.

Similar to diagnostic system, while concepts and constructs are essential for any clinical or therapeutic work, rigid adherence to them can be counter-productive. Once, I suffered from the concept of ‘castrating patient.’

## FACE, OR LOOSE OUT

As mentioned earlier, I was an active participant in therapeutic ward group meetings on the lines of therapeutic milieu. The participants were patients, nurses, doctors, consultants, and at times, even patients’ key-relatives. After a year, I started conducting weekly-once ward group meeting on my own in a ‘sub-acute’ ward, consisting of only patients and myself. I discussed the proceedings of each meeting with the consultant. After a few weeks, when a meeting was about to end, one patient said, “you are no good,” and another joined, “you are useless.” I was stunned, and momentarily lost my bearings, but, some how managed to say “we will discuss this issue in our next meeting.” I continued to be preoccupied with this experience, and was sleepless for two days.

After four days, while discussing about this session with the consultant, I expressed my preference that either the consultant attends the meeting with me to ‘buffer the crisis’ (‘bail-me-out’), or gets another registrar to take-over the group. The consultant's advice was stern: “Go and face the group. Otherwise, you will never be able to face a group again in your life.” He suggested a few alternative strategies to adapt in the next meeting. I did attend it, invited discussion about my conduct and role in the group. I was astonished to find the same two patients extremely supportive and insightful in the discussions. When that meeting ended, I felt extremely confident of my self, that ‘I can manage any group any where.’ Then, ***I lost absolute faith in the ‘castrating patient.’** I do not mean that such a phenomena does not exist. It had happened to me! I mean that it should eventually be possible to successfully ‘wade’ through any therapeutic situation, however unpleasant. The blunt message is: therapist's despair is suicidal to further learning. Be hopeful and think positively. There is always a way out!* Rigid adherence to any concept or construct can be dangerous.

*At a personal level, patients are not hostile to therapists; they dare not be so to their benefactor!* Negative-feelings emerging in therapy are part of the therapeutic process. Therapist has to make positive use of them so that both patient and therapist learn from it. Believe in the ancient wisdom, “whatever happens is for the good.” There may however be exceptions, as in the case of patients with active and violent paranoid delusions.

In this context, it is necessary to underscore a point: *Do not be afraid of making mistakes,* while at the same time, being cautions not to commit known mistakes. Fear of making mistakes prevents useful learning, and perpetuates wrong learning. There are too many variables operative, most of them un-known, and it is impossible to negotiate an ‘error-free therapeutic conduct.’ The only insurance against consequences of ‘mistakes’ is the triad of sincerity (honesty), genuinity (absence of pretence), and empathic good-will.

During that period, I was fortunate to recognize another concept, the professional's self-concept of “professional-ego” that could perhaps, ‘castrate’ further learning if given a free reign.

## PATIENTS CAN GET WELL WITHOUT ME !

A patient whom I followed-up regularly was an unmarried man in his thirties, living alone and working as a fitter in a factory. His diagnosis was ‘paranoid depressive psychosis.’ His delusions were centered around a perambulator in his back yard. It was the perambulator in which his grand mother used to take him out daily after he was orphaned early in his childhood. Even though manageable and functioning most of the time, occasionally he used to lapse into acute excarbations. At such times, he requied admission and ECTs. Over a period of time, I began to dread the prospect of seeing him. Every encounter with him was a reminder of my clinical impotence in bringing about any further improvement in his clinical state.

During one such follow-up review, I was very surprised to see him happy and smiling. I had never seen him smiling earlier, and for those readers who have read P.G.Wodehouse, ‘my jaw fell with a thud!’. I greeted him, “I am glad to see you well and happy. I am interested to learn how this excellent change came about.” He reported: (i) a few days ago he happened to cry in the factory, (ii) the personnel-officer took him to his office and enquired, (iii) he cried out his fears and worries relating to the perambulator, (iv) the personnel-officer drove the patient home and took away the perambulator, and (v) his fears and worries ceased, and depression disappeared! This account was a terrible shock to me and an affront to my professional ego. Momentarily, I even felt angry, “how dare he get well with out my help?” Of course, a few months later, his symptoms recurred; but, were milder.

***I lost absolute faith in the indispensability of mental health profession(al).** A patient can get well, and survive by means and methods other than offered by mental health professional! Also, the patient is capable of and responsible for doing so. If so, ‘why has he come to consult?’* ‘Why the patient has chosen not to put these abilities to use?’ Psychotherapeutic effort with any patient should attempt to answer this question. *It is this attempt that contributes to favorable therapeutic outcome.* Psychotherapy happens while the therapist is attempting to understand ‘why this patient has not been able to manage his or her problems?’ But, the outcome can be extremely variable.

*Sincerely believe that patient is capable of getting well and surviving by means and methods other than offered by mental health profession. Actively and positively, give responsibility to patient.* Of course, this principle may not apply in its entirety in such cases as mental retardation, etc.

*Professional or personal pride (as different from ‘self-respect’ and ‘confidence’) is a serious handicap to learn and practice psychotherapy.* I believe that *effective and continued learning* requires a state analogous to humility, something like: ‘I do not know, I want to know….“ In contrast, professional pride is a characteristic of one who knows!

## SWAMI VIVEKANADA'S CANNONS

In this context, it is interesting to consider what swami Vivekananda has said about teaching and learning. Here, we have to remind ourselves, that in a therapeutic situation bilateral ‘teaching-learning’ is taking place between the patient and the therapist. The cannons are:

- “Real teaching is: teaching how to learn. The student will then do his own learning.” In psychotherapeutic setting, best therapeutic benefit is when the patient learns how to cope. The next cannon gives clue to how this can best happen.
- “Best teaching is when the teacher is not aware he is teaching, and best learning is when the student is not aware of being taught.” While applying this cannon to psychotherapeutic situation, two questions pose themselves: (i) When is the therapist unaware of ‘doing psychotherapy or teaching patient how to cope?’ (ii) When is the patient unaware of being ‘taught or, helped?’ The answers are: The therapist is unaware of doing psychotherapy when he or she is busy understanding the patient. Even interpretations are only meant to lessen resistances and/or ‘loosen-up’ further abreaction or inflow of information. The patient is unaware of ‘learning to cope’ when he or she is busy interacting with and responding to the therapist.

Consequent to the above points, a few corollaries emerge:

- *Psychotherapy is not done, but happens. It happens when an individual's distress is favorably modified by interaction with another individual.* It happens when there is concern for another's distress, empathy, good-will, intent to relieve distress, and hopeful and sincere effort. These are ‘desirable therapist qualities’ advocated by Rogerians.
- As said earlier, *patient benefits not by therapist's objective of doing psychotherapy and consequent behavior, but by his/her objective of understanding: why, how, under what circumstances, and for what reasons is the patient in distress?* Consciously, or un-consciously, in a parallel fashion, the patient too will be following this ‘understanding’ and learns coping strategies in the bargain.
- *Psychotherapeutic benefit is a consequence of desirable therapist qualities. Without these, mere methods, techniques, and concepts are useless*. Therefore, these qualities should ideally become focus of training in psychotherapy. Even if they belong to the phenomena of personality, I believe that they can be cultivated and/or strengthened.

Now, it is very easy to appreciate how the pride of being a therapist (a product of professional-ego), of being a ‘guru,’ or of ‘doing psychotherapy’ can be a serious obstacle to therapeutic benefit.

## THERAPIST AND HIS TOOLS

I now shift the time and place to eighties and nineties at NIMHANS. From 1983 to 1994, I was involved in the department's psychotherapy training programme for psychiatry residents, and in evaluating their therapeutic performance. During this period, about 250 residents were trained and assessed. The department's effort was to offer a uniform training with the help of: (i) a structured format of training and supervision, both theoretical and supervisory, (ii) weekly meeting of the supervisors to exchange experiences in their weekly-once supervision-sessions, and (iii) structured assessment of the residents' skills at the end of each course. There were three important variables in the training programme: the supervisor, the resident, and the patient.

Over the period, my and fellow supervisors' cumulative observation was: (i) In spite of fairly uniform training procedure, there were individual variations in supervisory sessions. (ii) The residents, varied widely in how they conducted themselves in therapy sessions with their patients, in respect of concepts, methods and techniques used. (iii) Every resident was successful with some patients, unsuccessful with a few, and with variably moderate results with others. ***I lost absolute faith in methods and techniques.** It is not that they are useless; on the contrary, they are the tools of therapeutic management; but, they are less important than the person who uses them. They are useful only to the extent that their user makes them useful!* In a way, a therapist is to therapy what a director (*‘Sutradhari’*) is to a play. The therapist plays a major role in the therapeutic outcome.

Apart from the above events, there were also many, less dramatic events and, perhaps many more events that did not register in my conscious awareness that contributed to my learning.

## TOO MANY VARIABLES (OR, “TOO MANY COOKS”)

**Figure 1:**
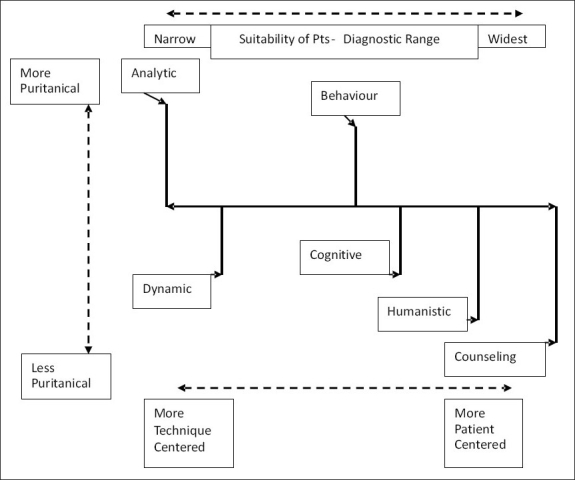
Continua in psychotherapeutic methods

There are many variables operative in psychotherapy. Therapist's personality and choices of concepts and techniques contribute to a continuum of therapeutic methods at least in two dimensions, as figuratively represented in Figure 1.

Then, there are variables that influence the psycho-therapeutic outcome. The important ones are: (i) Patient. (ii) Diagnosis. (iii) Socio-environmental context. (iv) Therapist. Consequently, the outcome necessarily follows Guassian distribution on each of the four dimensions. Therefore, absolute therapeutic effectiveness is a myth. The logical consequences of this fact are shown in Table 1.

**Table 1:** Therapist-effectiveness and patient-potential to benefit

Therapist-effectiveness	Patient-potential to benefit
Can benefit any patient (Very rare)	Can benefit from any therapist (Very rare)
Can benefit most patients	Can benefit from most therapists
Can benefit particular types of patients	Can benefit from particular types of therapists
Cannot benefit any patient (Probably, not true)	Cannot benefit from any therapist (Probably, not true)

But, it is essential to remember our ancient tenet that no sincere effort ever gets wasted. Even if the patient and therapist are unaware of it, even if there is no manifest therapeutic benefit, the patient carries forward ‘credits’ from previous therapy or therapies to reap the benefit with some later therapist!

## LEARNING FROM INDIAN MYTHOLOGY

In 1980s, when I became interested in collecting concepts relating to mental health in Indian mythology and related literature, I noticed a few shifts in my therapeutic orientation, which may appear radical in some respects.

1. A chapter (no. 4) titled ‘Psychotherapeutic Paradigms from Indian Mythology’ that I wrote for *Therapeutic Use of Stories* (Ed. By Kedar Nath Dwivedi, 1997, Routledge, London) describes the first of the effects. I was convinced that components of ideal human behavior are almost same as those of desirable therapist qualities, as well as those that correlate with state of well-being. That is, an individual who has components of ideal behavior as a part of personality will not only be inherently mentally healthy, but will also have a potential to be a ‘natural’ psychotherapist.
2. The second was a conviction that: as illnesses and difficulties (miseries) in human life are inevitable, a state of health is not defined by their absence, but by the individual's ability to effectively manage these inevitable challenges.
3. Consequent to the second, the third was a conviction that mental health is not a passive state, but has to be actively earned by facing the challenges, bearing the difficulties, managing the adversities, gracefully accepting un-avoidable limitations and failures, and re-attempting as appropriate, etc.
4. The fourth was a conviction that a **set of dynamic factors** that are opposites of components of ideal behavior can explain why and how individuals suffer psychologically. The type of suffering (viz. diagnosis) is dependent on pre-morbid-personality, nature of stress, and related factors. *These factors are also dynamic forces similar and parallel to the traditional ‘psycho-dynamics’ that we are usually familiar with. By keenly studying any detailed clinical-psycho-social case-history, it is possible to explain the patient's suffering as a consequence of aberrations in one or more of these factors.*

*These factors can be called **‘Humanistic Factors,’*** and can be used to explain any individual's psychopathology, in the same way as one uses classical psychodynamic theories. Moreover, these factors can complement other existing theories. The Humanistic Factors are:

- *Excessive or abnormal selfishness* that encroaches upon and obstructs one's civic, social, and other roles and responsibilities. It can contribute to such motivating forces as greed, competitiveness, intolerance, un-attainable ambitions and expectations, aggression, and inter-personal strife. This factor can reach insatiable proportions.
- *Excessive or abnormal pride,* which can also lead to consequences similar to that of selfishness. This can also reach insatiable proportions.
- *Not wanting to ‘pay the price,’ not wanting to ‘sacrifice,’ not wanting to put in necessary hard work or bear the necessary burden.* For example, let us assume the known fact that mental health is intimately related to ‘adjustment’ to one's needs, abilities, stresses, strains, and demands of living. What else is adjustment if it is not: (i) giving up some advantage for some other advantage, (ii) accepting and bearing some disadvantage in order to avoid some other disadvantage, (iii) giving up some goal/objective for some other goal, and (iv) accepting and tolerating some degree of unpleasantness, defects, burdens, etc. People suffer and make others also miserable because they do not want to sacrifice; they want every thing, with little or no effort.
- *Pretence (lack of genuinity, or hypocrisy)*. For example, pretending to be: (i) what one is not, or (ii) in a psychological state different from what one is in, or (iii) having an ability one does not have. Pretence, like acting, is extremely stressful. This factor is most probably, a consequence of the above three factors.
- *Dis-honesty*. This includes unfairness, exploitation, and not applying to oneself the same principles that one expects from others, etc. This factor is most probably, a consequence of the above four factors. The behavior of *lying* is related to this factor. Technically, any distortion, exaggeration, or denial, etc. of a known fact constitutes a lie. This is so even if such a process take place intra-psychically.
- *Desire or addiction to either sensation-seeking (thrill, or excitement seeking), or dulling or altering the conscious state for the purpose of avoiding the responsibility of experiencing the current state of being*. It is interesting that our pharmacological mode of management does some thing similar under the control of the therapist!

*It is frightening that in the present, modern culture, the entire media are brain-washing the population to cherish and imbibe the first four factors mentioned above. As a consequence, inevitably, they are forced to develop the last two factors also in the bargain.* Every one of us is a witness to this phenomenon.

[Ed: The concluding part of this series will appear in the next issue]

